# Measurement properties and confirmatory factor analysis of the Jefferson Scale of Empathy in Italian medical students

**DOI:** 10.1007/s40037-014-0137-9

**Published:** 2014-08-08

**Authors:** Paolo Leombruni, Mariangela Di Lillo, Marco Miniotti, Angelo Picardi, Guido Alessandri, Chiara Sica, Francesca Zizzi, Lorys Castelli, Riccardo Torta

**Affiliations:** 1 Clinical Psychology and Psycho-Oncology Unit, ’Rita Levi Montalcini’ Department of Neuroscience, University of Turin, 15, Via Cherasco, 10126 Turin, Italy; 2 Azienda Ospedaliera Marche Nord, Fano, Italy; 3 Mental Health Unit, National Center of Epidemiology, Surveillance, and Health Promotion, Italian National Institute of Health, Rome, Italy; 4 Department of Psychology, ‘La Sapienza’ University of Rome, Rome, Italy; 5 Department of Psychology, University of Turin, Turin, Italy

**Keywords:** Empathy, Medical students, JSE

## Abstract

Medical educators agree that empathy is essential for physicians’ professionalism and most studies on the patient-physician relationship demonstrate that this attitude has a key role in improving clinical outcomes. Literature findings show conflicting views in defining and measuring empathy. Nevertheless, the Jefferson Scale of Empathy (JSE) is a psychometric tool now widely used. Therefore, the aim of this study was to examine psychometrics and confirm factor structure of the Italian version of the JSE in Italian medical students (JSE S-Version). During 2012, 257 second-year Italian medical students completed the JSE S-Version. Internal consistency and test-retest reliability were assessed. A confirmatory factor analysis was performed to test the factor structure. The Italian JSE S-Version showed an acceptable internal consistency (r = 0.76) and test-retest reliability (r = 0.72). Confirmatory factor analysis found that the factor structure proposed by the developers of the tool provides an acceptable data fit. In this sample, female medical students showed a higher mean empathy score than did males. The present study provides evidence confirming the structural validity and reliability for the Italian JSE S-Version. Further studies are needed to confirm these findings and to explore cross-cultural differences and their implications.

## Introduction

Despite the difficulties in precisely defining empathy, almost all agree that empathy plays a key role in positive doctor-patient relationships and contributes to improvements in clinical outcomes [1]. Consequently, empathy should be an issue specifically addressed in the core curriculum of medical studies, together with communication skills. Both have to be considered essential professional characteristics for a good physician and, in general, for all helping professions [2]. Therefore, to test the effectiveness of educational programmes, it is necessary not only to define *the meaning* of physician empathy, but also *how to measure* it. In fact, despite the wide literature regarding what physician empathy is, there is still not a generally accepted clear definition of this concept.

Aring [3] and Rogers [4] and, more recently, Hojat [5] described empathy as an ability to perceive the internal frame of reference of another person and to appreciate his or her feelings.

Many researchers agree that empathy is an important attribute for the physician because it helps to better understand, and to communicate with the patient.

Hojat and colleagues postulated that physician empathy has two components: an affective and a cognitive one [1], and Mercer and Reynolds proposed an integrative approach where physician empathy can be considered both a multidimensional and a skill-based construct [6]. On the other hand, there are authors who specify that physician empathy is more a learnable professional skill than an innate personality trait [7].

Therefore, many standardized instruments have been developed [8, 9] to assess levels of empathy. One of the most used tools that consider the point of view of physicians is the Jefferson Scale of Empathy (JSE), created by researchers at Jefferson Medical College in the US [5, 10–12]. These authors proposed physician empathy as ‘predominantly a cognitive attribute (whereas sympathy is primarily an ‘affective’ personal quality)’ that ‘involves an understanding of the inner experiences and perspectives of the patient… with a capacity to communicate this understanding’ and combined with an intention to help the patient [5, 13]. The JSE was specifically developed to measure empathy among physicians and medical students to ‘capture the essence of an empathic relationship in patient care situations’ [5]. It has been translated and validated in several countries all over the world [13]. In Italy, it has already been tested in a sample of physicians [14] but never among medical students.

The present effort to spread the use of the tool is important when considering the emerging conflicting results about the evolution of empathy toward the course of graduation [15] and the possible influence of cross-cultural elements in explaining at least some of the differences observed.

The present study was designed to test three research hypotheses:

1. The Italian version of the JSE is a reliable psychometric tool and its factor structure is consistent with the model proposed by the developers of the scale;
2. Females exceed males in the JSE score;
3. Cross-cultural differences emerge in the JSE scores.

Subsequently, the aims of the present study are twofold:

1. To examine the psychometrics of the Italian version of the JSE in a sample of Italian medical students by verifying its internal consistency, test-retest reliability and factor structure;
2. To assess Italian medical students empathy level.

## Methods

### Participants

The research participants were undergraduate (second-year) medical students who attended the University of Turin Medical School. Ethical approval was obtained from the University of Turin Ethical Review Committee. The study was conducted in accordance with the latest version of principles of the Declaration of Helsinki.

### Instrument

The JSE is a self-report paper and pencil questionnaire developed to assess medical students’ attitudes toward empathic engagement in the context of patient care. It consists of 20 items, each answered on a seven-point Likert-type scale. Half of the items are positively worded (directly scored as follows: 1 = strongly disagree, 7 = strongly agree) and half are negatively worded (inversely scored as follows: 1 = strongly agree, 7 = strongly disagree). The possible total score ranges from 20 to 140. Higher values indicate more positive attitudes toward empathic patient care, hence a higher degree of empathy [13].

The original scale was developed to be administrated to medical students; it was called the Jefferson Scale of Physician Empathy and so the acronym was JSPE [10, 11]. But, due to the increasing popularity of the JSPE and the growing demand for its use in different contexts, the authors of the scale renamed it ‘Jefferson Scale of Empathy’ (JSE). Nowadays, the JSE can be administered (with appropriate slight modifications of some items) to physicians and other health professionals (JSE HP-Version), medical students (JSE S-Version) and students in health professions other than medicine (JSE HPS-Version) [13]. The Italian translation and the preliminary psychometrics of this tool were performed in a previous study conducted among a sample of Italian physicians [14] using a version designed specifically for physicians and other health professionals derived from the original JSPE [11].

For the present study, we used the JSE S-Version in its Italian translation provided by Maio and Louis, both medical education researchers of Jefferson Medical College [13].

### Procedure

Questionnaires were administered to students at the beginning of their second year of medical training, after a brief description of the study during class time. To evaluate test-retest reliability of the JSE S-Version, a subsample of the participants were retested after 2 weeks. This interval was chosen as an acceptable compromise between recall effects and unwanted condition changes. Responses were confidential and collected anonymously (students identified themselves using nicknames). Each participant received an identification number when the data were entered.

### Statistical analyses

SPSS for Mac, version 20.0, was used to calculate descriptive statistics, to perform the Student’s *t* test and univariate analysis of variance for examining between-group differences in the JSE S-Version scores, to calculate the internal consistency as measured by Cronbach’s alpha, and to compute the test-retest reliability as measured by the intra-class correlation coefficient between scores on the first and the second administration of the questionnaire. All statistical tests were 2-tailed, with alpha set at 0.05.

Confirmative factor analysis was conducted using Mplus 4.0 [16]. This is a form of factor analysis used to test whether observed data fit a postulated measurement model. As opposed to a theory-generating method such as exploratory factor analysis, confirmatory factor analysis is a theory-testing model, as the researcher begins with a hypothesis prior to the analysis. This model, or hypothesis, specifies which variables will be correlated with which factors and which factors are correlated. The hypothesis is based on theory, previous research, or both. Given that the predetermined model specifies the number and composition of the factors, the research is able to explicitly test hypotheses regarding the factor structure of the data. In the present study, following procedures suggested by Hojat and colleagues [11] and by Shariat and Habibi [17], we compared the fit of three models. A one-factor model (M1) in which all 20 items were forced to load on a single factor labelled ‘general medical student’s empathy.’ An orthogonal three-factor model (M2), entailing the ‘perspective taking,’ ‘compassionate care,’ and ‘standing in the patient’s shoes’ factors posited as uncorrelated. Finally, a three-factor oblique model (M3), entailing these last factors posited as correlated.

To deal with the ordinal nature of the data, the estimator used for the analysis was a mean- and variance-adjusted least-squares estimator (weighted least squared mean variance). Furthermore, as the normality assumption was non-tenable (Table 3), in all successive models we employed the Satorra-Bentler [18] scaled Chi square (*χ* ^2^) statistic and corrected standard errors. This correction takes into account the deviation from normality observed in the data.

All models were specified using delta parameterization [16], and their fit was evaluated by means of the following overall indexes: *χ* ^2^ statistic, the root mean square error of approximation, the comparative fit index, and the weighted root mean square residual. Recommended cut-off points for the root mean square error of approximation are 0.08 [19] or 0.06 [20], for the comparative fit index 0.90 [21] or 0.95 [20]. For the weighted root mean square residual, the cut-off of 1.0 has moderate to strong power to detect misspecified models with acceptable Type I error [22]. Yu reported that the weighted root mean square residual, similar to the *χ* ^*2*^, might be too powerful for trivial misspecification on factor covariance when sample size is large [22]. Since the factor models are truly nested models, their relative fit was compared using the scaled difference *χ* ^2^ devised by Satorra and Bentler [23].

## Results

### Response rates

Among a total population (*n* = 390) of second-year medical students of the University of Turin Medical School who were invited to participate, 76.9 % (*n* = 300) agreed to do so and 257 returned the correctly completed JSE questionnaire adapted for medical students (JSE S-Version), for an overall response rate of 65.9 %. No demographic differences were found between students who did and did not return or complete the questionnaire. All participating students voluntarily agreed to participate in the study and were properly informed about its purposes and methods. Students did not receive any incentive for their participation in the study.

### Demographics

The sample considered in statistical analyses (*n* = 257) included 114 (44.4 %) males and 143 (55.6 %) females who returned the correctly completed JSE-S Version. The mean age of the overall sample was 20.6 years (SD = 20.7). All students were Caucasians.

### JSE S-Version scores and gender differences among Italian medical students

For questionnaire completion, no time limit had been set but students took roughly 5-10 min to complete it. The mean total score of the JSE S-Version was 108.71 (SD = 10.60). Female students scored significantly higher than male students, providing small effect size (*d* = 0.32). Tests of between-subject effects performed on the three factors of the scale showed that females scored significantly higher on the ‘compassionate care’ factor, while the difference was not significant for the factors ‘perspective taking’ and ‘standing in the patient’s shoes’. The between-group differences, despite being significant due to the large sample size, were small in absolute terms (Table 1).

**Table 1:** JSE S-Version scores

JSE S-Version subscales and total score		Sample *N* = 257	Males *N* = 114	Females *N* = 143	*p* value (males vs. females)
		M ± SD	M ± SD	M ± SD	
F1. ‘Perspective taking’	m.r. †	5.56 ± 0.66	5.50 ± 0.58	5.61 ± 0.72	0.187
	s.r. ‡	55.60 ± 6.65	54.97 ± 5.84	56.11 ± 7.21	0.173
F2. ‘Compassionate care’	m.r. †	5.66 ± 0.65	5.53 ± 0.63	5.75 ± 0.66	0.007*
	s.r. ‡	45.25 ± 5.22	44.27 ± 5.03	46.03 ± 5.26	0.007*
F3. ‘Standing in the patient’s shoes’	m.r. †	3.92 ± 1.24	3.79 ± 1.15	4.03 ± 1.30	0.123
	s.r. ‡	7.85 ± 2.47	7.59 ± 2.30	8.06 ± 2.59	0.126
JSE S-Version total score	m.r.†	5.44 ± 0.53	5.34 ± 0.46	5.51 ± 0.57	0.010*
	s.r. ‡	108.71 ± 10.60	106.83 ± 9.10	110.21 ± 11.47	0.011*

* α set at 0.05; 255 degrees of freedom; † mean ratings; ‡ sums of ratings

Statistics at item level are available for the interested reader.

### Reliability

Internal consistency was calculated by Cronbach’s alpha. Reliability for the entire scale was 0.76 for the overall sample, 0.73 for males and 0.79 for females. The test-retest reliability of the JSE S-Version as measured by the intra-class correlation coefficient was 0.72 (95 % CI 0.63–0.79), calculated on 153 (59.5 %) students (73 males, 80 females).

### Confirmatory factor analysis of the Italian translation of the JSE S-Version

The model fit indices of the one-factor and the orthogonal and correlated three-factor models are reported in Table 2. Results showed that the oblique three-factor model provided an acceptable data fit, while the one-factor model and the orthogonal three-factor model revealed a poor data fit.

**Table 2:** Parameter estimates from confirmatory factor analysis of the oblique three-factor model

JSE S-Version items	M ± SD	s	k	H	S & H	λ
Factor 1. Perspective taking						
Item 16. Physicians’ understanding of the emotional status of their patients, as well as that of their families, is an important component of the physician-patient relationship	6.12 ± 0.88	−0.96	0.05	0.70	0.62	0.70
Item 13. Physicians should try to understand what is going on in their patients’ minds by paying attention to their non-verbal cues and body language	6.01 ± 1.05	−2.50	9.15	0.62	0.51	0.74
Item 20. I believe that empathy is an important therapeutic factor in medical treatment	5.87 ± 1.11	0.27	−0.57	0.60	0.64	0.80
Item 15. Empathy is a therapeutic skill without which the physician’s success is limited	4.98 ± 1.46	−1.30	1.71	0.58	0.64	0.59
Item 10. Patients value a physician’s understanding of their feelings which is therapeutic in its own right	6.00 ± 1.05	−0.39	−0.60	0.58	0.58	0.60
Item 2. Patients feel better when their physicians understand their feelings	6.30 ± 1.00	0.29	−0.83	0.50	0.49	0.43
Item 4. Understanding body language is as important as verbal communication in the physician-patient relationship	6.07 ± 1.07	−2.50	9.20	0.46	0.54	0.49
Item 9. Physicians should try to stand in their patient’s shoes when providing care to them	5.15 ± 1.49	−1.20	1.41	0.46	0.54	0.33
Item 5. A physician’s sense of humour contributes to a better clinical outcome	4.64 ± 1.59	−0.65	−0.22	0.45	0.44	0.30
Item 17. Physicians should try to think like their patients in order to render better care	4.46 ± 1.58	−1.45	3.28	0.46	0.35	0.30
Mean loadings factor 1				0.54	0.53	0.53
Factor 2. Compassionate care						
Item 11. Patients’ illness can be cured only by medical treatment; therefore, physicians’ emotional ties with their patients do not have a significant influence in medical or surgical treatment	5.88 ± 1.21	−1.48	2.59	0.60	0.70	0.67
Item 8. Attentiveness to patients’ personal experience does not influence treatment outcomes	5.74 ± 1.19	−1.42	2.71	0.59	0.28	0.49
Item 7. Attention to patients’ emotions is not important in history taking	6.46 ± 0.87	−1.29	1.94	0.55	0.59	0.70
Item 14. I believe that emotion has no place in the treatment of medical illness	6.12 ± 1.14	−1.64	2.87	0.50	0.69	0.71
Item 18. Physicians should not allow themselves to be influenced by strong personal bonds between their patients and their family members	3.27 ± 1.44	−0.45	−0.51	0.44	0.06	0.04*
Item 1. Physicians understanding of their patients’ feelings and the feelings of their patients’ families does not influence medical or surgical treatment	5.38 ± 1.55	−1.26	2.89	0.43	0.36	0.33
Item 19. I do not enjoy reading non-medical literature or experiencing the arts	6.43 ± 1.12	−0.28	−0.65	0.37	0.40	0.31
Item 12. Asking patients about what is happening in their lives is not helpful in understanding their physical complaints	5.98 ± 1.11	0.32	−0.45	0.37	0.53	0.64
Mean loadings factor 2				0.48	0.43	0.49
Factor 3. ‘Standing in the patient’s shoes’						
Item 3. It is difficult for a physician to view things from patients’ perspectives	4.07 ± 1.35	−2.47	6.40	0.74	0.65	0.79
Item 6. Because people are different, it is difficult to see things from patients’ perspectives	3.78 ± 1.49	−1.07	1.08	0.66	0.55	0.69
Mean loadings factor 3				0.70	0.60	0.74

*Notes* All parameter estimates were significant at *p* < 0.05, except item 18 (*); the items are numbered according to Shariat & Habibi (2012)

*s* skewness, *k* kurtosis, *H* standardized factor loadings from Hojat and colleagues (2002b), *S & H* standardized factor loadings from Shariat & Habibi (2012), *λ* standardized factor loadings for the present study

In Table 3, where the three models are directly compared with each other, data showed that the oblique three-factor model performed better than the two other models. The model fit improved when the residual correlation between items 17 and 19 was excluded (*χ* ^*2*^ = 407.55, df = 166; *p* < 0.01; comparative fit index = 0.944, root mean square error of approximation = 0.075, weighted root mean square residual = 0.959). Under this best fitting model, correlations between latent factors were 0.24 (‘perspective taking’ with ‘compassionate’ care), 0.73 (‘perspective taking’ with ‘standing in the patient’s shoes’) and 0.26 (‘compassionate care’ with ‘standing in the patient’s shoes’). Table 2 shows standardized factor loadings. With regard to the ‘perspective taking’ factor, all loadings were significant and ranged from 0.30 to 0.80 (mean = 0.53; SD = 0.18). For the ‘compassionate care’ factor, except the loading of item 18, all loadings were significant and ranged from 0.31 to 0.71 (mean = 0.55; SD = 0.26). Finally, the two items pertaining to the ‘standing in the patient’s shoes’ factor displayed significant and high loadings on their factor.

**Table 3:** Goodness-of-fit indexes for the three alternative confirmatory factor analysis models

	*χ* ^*2*^	*df*	CFI	RMSEA	WLRM
M1	1548.53*	171	0.68	0.18	0.19
M2	1887.89*	170	0.50	0.20	2.32
M3	581.16*	167	0.91	0.08	0.99

	Δ*χ* ^*2*^	Δ*df*	*p*
M2 vs M1	310.90	1	<0.01
M3 vs M1	1289.90	4	<0.01
M3 vs M2	901.01	1	<0.01

*M1* one-factor model in which all 20 items were forced to load on a single factor labelled ‘general medical student’s empathy’, *M2* orthogonal three-factor model entailing the ‘perspective taking’, ‘compassionate care’ and ‘standing in the patient’s shoes’ factors posited as uncorrelated, *M3* oblique three-factor model entailing these last-mentioned factors posited as correlated, *χ* ^*2*^ the Chi square statistic, *df* degrees of freedom, *CFI* the comparative fit index, *RMSEA* the root mean square error of approximation, *WLRM*: the weighted root mean square residual, * *p* < 0.01

## Discussion

In summary, findings from the present study suggest that the Italian translation of the JSE S-Version, administered to undergraduate medical students, has satisfactory levels of internal consistency and test-retest reliability, corroborating the validity of the three-factor structure proposed by the developers of the tool [11]. Furthermore, female participants show higher levels of empathy than males.

Still regarding psychometric properties of the JSE S-Version, although its sensitivity to change needs still to be determined, its aforementioned reliability suggests that it may also have potential as an assessment tool to evaluate fluctuation in empathy over time or to verify the impact of specific training on levels of empathy.

Regarding the confirmatory factor analysis, the results of the present study showed that the oblique three-factor model suggested by the developers [11] provided an acceptable data fit, and the item loadings in the Italian sample were similar compared with the study by Hojat and his colleagues [11] and slightly higher compared with the study by Shariat and Habibi [17]. The evidence that the oblique model (where factors were allowed to be correlated) provided the best fitness could be consistent with the conceptual framework of a multidimensional notion of physician empathy. This construct seems to be composed by at least three dimensions, correlated to each other. ‘Compassionate care’ and ‘standing in the patient’s shoes’ could be considered more specific to the patient-physician relationship and linked to the intention to help someone, whereas ‘perspective taking’ could be considered relevant in every type of human interaction [11]. Given this construct, it is reasonable to assume that the empathic behaviour of a physician could emerge from the interconnection of these three cognitive attributes.

Furthermore, while our findings, overall, corroborate the structural validity of the instrument, some factor loadings of the Italian version were found to be low (item 5 and item 17) and very low (item 18). This suggests that these items may benefit from some degree of revision, as they do not seem to function optimally.

Looking at the findings of the total mean score of empathy from the student population already studied in different countries, the total mean score of empathy of the Italian sample is significantly higher than in the UK [24], Iran [17] and Japan [25] but lower than in the USA [26]. In order to better understand and properly discuss these differences, cross-cultural comparisons should be more extensively researched. However, this is beyond the scope of the present study.

In the present study, female students showed a significant but slightly higher empathy level than males. This difference seems to be determined in particular by their higher performance on the ‘compassionate care’ factor. In contrast, Shariat and Habibi [17] found that their observed gender difference was mainly due to the ‘perspective taking’ factor. These authors noted that the negatively worded items (present in the ‘perspective taking’ factor and absent in the ‘compassionate care’ factor) could somehow have an influence in revealing assumed gender differences. In literature, unfortunately, data are inconsistent: several studies did show females to have a higher score of empathy than males, both among physicians [11, 28] and students [17, 25, 29]. A few studies did not show any differences [14, 27].

### Limitations

The present study has some limitations that need to be underlined. First of all, the cross-sectional methodology, which gives an idea on the empathy level of the Italian students at the second year, does not allow to collect data on empathy trends. Future longitudinal studies are necessary to contribute to the current debate on the supposed ‘decline’ of this attitude [26]. The second relevant limitation regards the generalizability of the data. Although the demographics are representative of the Italian academic context, all the students recruited in the study belong to a single University site and fall within a single ethnic group, and this composition of the sample does not account for the possible differences among students from different regions of Italy or belonging to other ethnic groups.

## Conclusion

Despite the aforementioned limitations, the present study is the first, to our knowledge, to use the Italian JSE S-Version, providing psychometric information about this tool. In particular, the results show that this measuring instrument has a satisfactory internal consistency and test-retest reliability. Moreover, findings from confirmatory factor analysis corroborate the structural validity of the oblique three-factor model suggested by the developers [11]. Regarding the empathy level of the Italian medical students, females obtained higher empathy rates than males, as emerged in previous studies [17, 25, 29]. However, further studies are needed to confirm these preliminary results, and to better explore the impact of cross-cultural differences on empathy among this population.

## Essentials

- The Italian JSE S-Version seems to be a reliable psychometric instrument.
- The oblique three factor model proposed by the JSE S-Version developers seems to provide an acceptable data fit.
- The Italian JSE S-Version seems to show a structural validity; nevertheless, some items do not seem to function optimally.
- Female Italian medical students show slightly higher empathy than their male colleagues as measured by JSE S-Version.
- Italian medical students show higher empathy than UK, Iranian and Japanese medical students and lower than American as measured by JSE S-Version.
